# Enhancing Upper Secondary Students’ Situational Engagement and Cognitive Prerequisites of Learning Through the Physically Active Academic Lessons Intervention: Protocol for a Mixed Methods Cluster Randomized Individual Crossover Trial

**DOI:** 10.2196/84601

**Published:** 2026-02-03

**Authors:** Heidi J Syväoja, Susanna Takalo, Tuomas Kukko, Nina Salmela, Harto Hakonen, Janne Kulmala, Heidi Lindfors, Hermanni Oksanen, Pekka Räsänen, Kati Mäkitalo, Tuija H Tammelin

**Affiliations:** 1 Likes, School of Health and Social Studies, Jamk University of Applied Sciences, Piippukatu 2, Jyväskylä, Central Finland, FI-40100, Finland, (+358) 400248133; 2 Early Childhood and Music Education Studies (CHIMES), Faculty of Education and Psychology, University of Oulu, Oulu, Finland; 3 Learning and Learning Processes Research Unit, Faculty of Education and Psychology, University of Oulu, Oulu, Finland; 4 City of Oulu, Oulu, Finland; 5 Epilepsia Helsinki, Department of Pediatric Neurology, HUS Helsinki University Hospital, Helsinki, Finland; 6 Turku Research Institute for Learning Analytics, Faculty of Science, University of Turku, Turku, Finland

**Keywords:** physically active learning, physically active break, student engagement, executive functions, acute effects, experience, cluster randomized individual crossover trial

## Abstract

**Background:**

Internationally, physical activity is successfully integrated into academic lessons in primary schools, showing promising results on cognition and student engagement. However, there is a lack of knowledge about its effects and feasibility for individual situated learning processes in upper secondary school.

**Objective:**

This protocol describes the design and methods of the Physically Active Academic Lessons (PAAL) study, a mixed methods, cluster randomized, individual crossover trial. The PAAL study aims to examine the acute effects of physically active academic lessons on cognitive prerequisites of learning (alertness and executive functions) and situational engagement (behavioral, cognitive, and emotional engagement; disaffection; competence experiences; and help seeking), as well as factors modifying these effects (physical and mental load and perceived physical and academic competence). Further, subject teachers’ and students’ experiences and perceptions of physically active academic lessons in general upper secondary school will be explored.

**Methods:**

The first part of the PAAL study involves exploring subject teachers’ experiences of facilitators, barriers, usefulness, and the meaning of physically active academic lessons for the situational learning process through semistructured interviews with 14 teachers. The second part consists of a cluster‑randomized individual crossover trial including 168 students in mathematics and foreign language lessons, followed by interviews with 30 students.

**Results:**

Funding for the study was obtained in May 2023. Ethical approval for the teacher interviews was granted in September 2023, and for the student trial in December 2023. Data collection was completed between October 2023 and November 2024. Data analysis is ongoing. The findings of the study will provide essential evidence-based information on physically active classroom practices that support teachers and schools in implementing pedagogical methods that enhance student learning and well-being in upper secondary schools.

**Conclusions:**

The background, design, content of the intervention, and methods of the PAAL study are presented. This study aims to address a gap in the literature regarding the feasibility and effectiveness of physically active methods during academic lessons in upper secondary school.

## Introduction

### Background

In a well-functioning educational environment, young people are not only performing academically but are also satisfied with their lives and feeling good physically and mentally [1]. However, young people’s mental health disorders have been predicted to be a leading cause of disability in high-income countries [2]. For example, in the United States, rates of major depressive episodes increased by 52% from 2005 to 2017 among adolescents aged 12 to 17 years and by 63% from 2009 to 2017 among young adults aged 18 to 25 years [3]. Likewise, in Finland, a recently published student barometer shows a decline in upper secondary school student well-being, with up to 62% finding studying is mentally taxing [4]. School burnout can, at worst, lead to academic failure, school dropout, and other mental health issues [5,6]. In addition to mental well-being, the physical health and functional capacity of young people are also a concern, as globally about 80% of students aged 11 to 17 years do not engage in sufficient physical activity (PA) for their health [7]. In Finland, general upper secondary students aged 16 to 18 years remain sedentary for 9 to 10 hours per day [8], most of which occurs in educational institutions. Prolonged sedentariness is harmful to both physical and mental health and may interfere with learning. In contrast, increased PA positively impacts physical health, strengthens mental health, and promotes brain health, cognitive function, and academic performance [9].

Current research supports increasing PA during the school day, including during academic lessons in children [10-12]. In school-based interventions, PA has been implemented into academic lessons in various ways, such as through PA breaks and physically active learning (PAL) [12,13]. PA breaks are intended to interrupt prolonged sedentary behavior by increasing PA [14,15] and to create optimal conditions for learning after the break by enhancing cognition, such as attention and executive functions [16], as well as classroom behavior [12,14,15] and learning outcomes [16]. Executive functions, a group of higher-order cognitive processes involved in self-regulatory and goal-directed actions, include key components such as inhibition (eg, the suppression of inappropriate or distracting responses), working memory, and cognitive flexibility [17]. Executive functions are strong predictors of academic performance [18].

In addition to these effects, PAL has been shown to engage students in learning by increasing their enjoyment of the learning process [10,11] and enhancing memory recall by constructing rich and elaborative representations [19]. PAL increases PA without reducing active academic learning time [20], as PA is integrated into the academic learning content of subjects other than physical education [21]. Student engagement is one of the key learning prerequisites in terms of students’ involvement and commitment to school and their own learning [22,23], and can be seen as consisting of several components, such as behavioral (eg, student conduct and on-task behavior), emotional (eg, students’ emotions felt towards learning, teachers, and classmates), and cognitive engagement (eg, students’ motivational goals and self-regulated learning) [22,23], as well as social engagement [24]. Student engagement is context-dependent and can be promoted through internal and external changes in the context and environment [22,25].

### Prior Work

Preliminary findings suggest that the effects of acute PA on older adolescents’ and young adults’ cognitive functions [26,27] and student engagement [28,29] are similar to those in childhood. However, due to limited repeated studies, inconsistency, and low methodological quality of available evidence, the benefits of PA among older adolescents have not been confirmed in an academic context [14,30,31]. More evidence-based information is needed on how physically active approaches during lessons acutely affect the prerequisites of learning compared with typical academic lessons without PA. Furthermore, not all young people benefit from PA in the same way [31]. While late adolescence is a vulnerable period for coping with mental, physical, and academic demands [32,33], mental load, including stress and anxiety, has been shown to impair executive functions [34,35] and efforts to be physically active [36]. Furthermore, perceived physical and academic competence predict participation in PA [37,38] and learning outcomes [39], while previously acquired skills, such as motor competence, may modify the effects of PAL [40]. Therefore, physical and mental load, as well as physical and academic competence, may influence the effectiveness of physically active pedagogical approaches. Further research is needed to clarify the role of potential moderating factors in the outcomes of physically active academic lessons.

Teachers across all subjects play a crucial role in implementing PAL and PA breaks. However, many teachers are not trained to teach through PA and often possess limited knowledge and skills regarding physically active classroom practices [41]. Previous research suggests that teachers interpret PAL through the lens of their professional identity and established teaching practices [42]. Therefore, it is important to understand subject teachers’ long-term experiences of implementing PAL in authentic academic lessons. Most existing PAL research has focused on the perspectives of subject teachers working with pupils under the age of 16 years [41,43]. There is currently a lack of research on general upper secondary school teachers’ experiences regarding the implementation of PAL and PA breaks.

Learning is context dependent, yet only a limited number of studies have examined situational variation in student engagement. Research in lower and upper secondary education indicates that students respond to contextual changes. Therefore, it is important to explore students’ personal observations and experiences in upper secondary lessons [44] to gain a deeper understanding of how to design effective and sustainable interventions [45] that influence their behavior and engagement in certain situations. Elementary school students have found physically active breaks and PAL to be enjoyable and beneficial for learning, with breaks offering a refreshing pause from schoolwork and PAL creating engaging and meaningful learning experiences in primary and lower secondary education among children aged 7 to 15 years [46-49]. Recent studies by Fred et al [50] and Romar et al [51] are among the few that have explored upper secondary students’ experiences of PA breaks and PAL, which students report as a welcome change from sitting, enhancing concentration, supporting learning, and making lessons more enjoyable—thereby promoting engagement. However, because most evidence on classroom-based PA interventions comes from primary school settings, and the few upper secondary studies involve small samples with limited generalizability, further research is needed to amplify students’ voices and explore situational variation in engagement [44,51-53].

### Goal of the Study

The Physically Active Academic Lessons (PAAL) study evaluated teachers’ and students’ experiences and perceptions during physically active academic lessons in a general upper secondary school. Specifically, we investigated subject teachers’ perceptions and experiences regarding the usefulness of physically active classroom practices implemented in different ways throughout the academic year. This included both the benefits and challenges associated with PAL and PA breaks, experiences of applying physically active classroom practices during various phases of teaching within academic lessons, and perceptions of how these methods support the achievement of learning objectives across different school subjects. In addition, we investigated students’ perceptions and experiences on students’ situational engagement (behavioral, emotional, cognitive, and social engagement) and their relation to physically active academic lessons, as well as their views on the usefulness of PAL and PA breaks. Moreover, we examined the acute effects of PAL and PA breaks on students’ situational engagement and cognitive prerequisites of learning. We especially examined how PAL and PA breaks affect students’ alertness, executive functions, behavioral, cognitive, and emotional engagement, as well as disaffection, competence experiences, and help seeking during academic lessons compared to lessons without PA. Furthermore, we examined how current physical and mental load or perceived physical and academic competence modify the acute effects of PAL and PA breaks on situational engagement and cognitive prerequisites of learning.

## Methods

### Study Design

**Figure 1.**
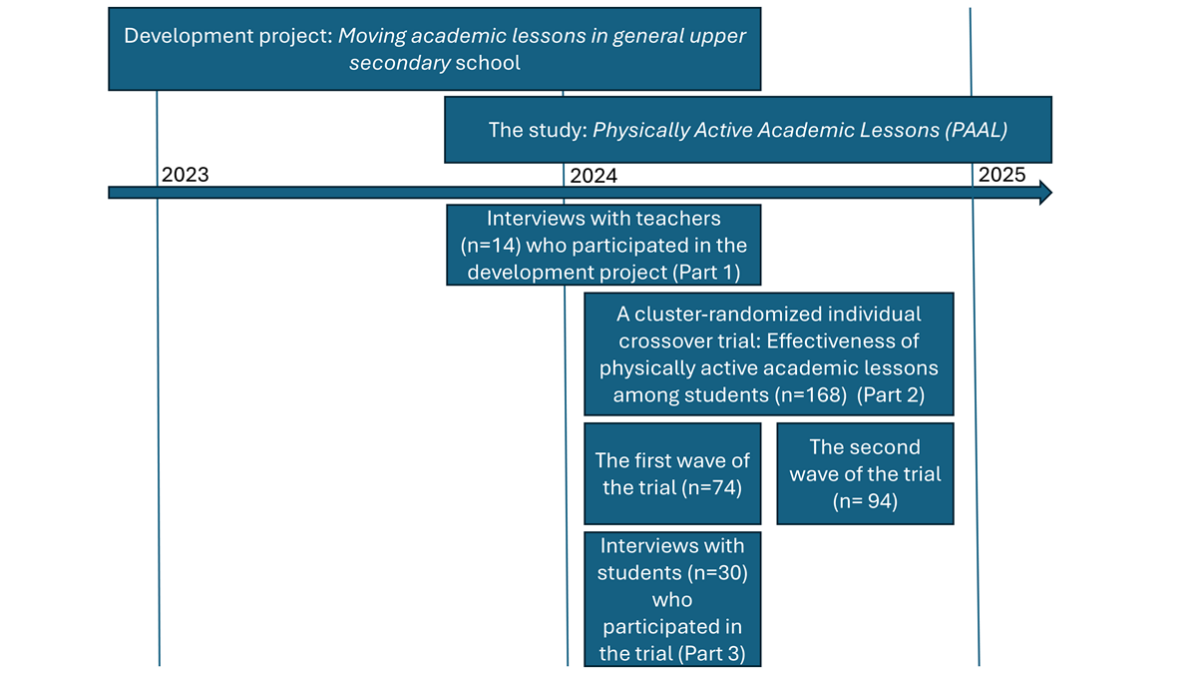
The overall study design of the Physically Active Academic Lessons (PAAL) study. The PAAL study consists of teacher interviews (Part 1), a cluster-randomized individual crossover trial among students (Part 2), and their interviews (Part 3).

This protocol (version 1) followed the Standard Protocol Items: Recommendations for Interventional Trials (SPIRIT) statement (see the SPIRIT checklist in Checklist 1) and describes the design and methods of the PAAL study. The PAAL study consists of teacher interviews (Part 1), a cluster randomized individual crossover trial among students (Part 2), and their interviews (Part 3). The overall study design is presented in Figure 1.

### Teacher Interviews (Part 1)

#### Content of the Development Project Before Teacher Interviews

The evaluation of subject teachers’ experiences and perceptions of physically active academic lessons in general upper secondary school was carried out in the context of the development project M*oving Academic Lessons in General Upper Secondary School* (2022‐2024). The aim of the development project was to promote the learning, well-being, and health of general upper secondary school students by combining PA with academic lessons. This development project was funded by the Ministry of Education and Culture and implemented for general upper secondary school teachers in Finland in cooperation with a national action program, Finnish Schools on the Move.

Fourteen subject teachers (n=14) from 8 different schools accepted an invitation to participate in the development project as PAL pioneer teachers in spring 2022. Participants represented 16 different teaching subjects, and their teaching experience in general upper secondary education ranged from 2.5 to 29 years, with a mean of 12 years. The subject teachers were asked to use and create a variety of teaching methods to suit PA with their own subject lessons’ topics and contents, and implement PA breaks during academic lessons. The development project was reviewed to inform the formulation of teacher interview questions (Part 1 of the PAAL study) and the planning of teaching methods for the intervention (Part 2 of the PAAL study).

#### Participants

Fourteen PAL pioneer teachers involved in the development project were invited to participate in the PAAL study teacher interviews conducted between October 2023 and May 2024, and all invited teachers (13 females and 1 male; ages 30‐62 y) accepted the invitation. Participants received correct and sufficient information about the study, including its objectives and the intended use of the interview data.

#### Ethical Considerations

Participation was voluntary, and participants were informed of their right to withdraw from the study at any time. Written informed consent was obtained from all participants prior to the interviews. Privacy and confidentiality were assured, and no financial or other compensation was offered. All identifying details have been omitted from the published written descriptions to protect the anonymity of the participants.

Before data collection (September 2023), the need for an ethical review of this part of the study was requested from the Ethics Committee of Human Sciences of the University of Oulu. According to the statement of the Ethics Committee of Human Sciences of the University of Oulu, an ethical review is not required when research involving human participants complies with the 2019 guidelines of the Finnish National Board on Research Integrity (TENK) [54]. This study was conducted in accordance with TENK’s guidelines. In addition, before teacher data collection, the study was approved by the Education and Culture Services of the municipality where the data were collected (OUKA/11121/07.01.04.02/2023) and fulfilled the ethical standards for empirical research.

#### Methods for Teacher Interviews

The participating subject teachers were interviewed after implementing PAL and PA breaks in their teaching for more than 12 months. The interview questions were finalized after a pilot interview. The list of final interview themes was shared with participants 2 days before the interview. Interviews were conducted at times and locations convenient for the teachers: at the participant’s school, at the university, or at the interviewer’s home. Teachers were asked to describe their experiences with PAL and PA breaks. The questions were organized around the main themes (see *Teacher Interviews* in Multimedia Appendix 1), which were developed through discussions with PAL pioneer teachers and reflections from the development project. In addition, similar overarching themes have been identified to summarize teachers’ perspectives on implementing PAL in primary schools [55]. Although the interviews adhered to the main themes, the order of questions varied between interviews based on the flow of the conversation between the interviewer and the interviewee. The semistructured interviews were recorded and transcribed during the autumn of 2023 and the spring of 2024 in Northern Finland. Interviews lasted 63 to 130 minutes (mean 95 min) and were done individually. Together, there were 365 pages of long transcribed text. PAL and PA breaks were treated as distinct classroom activity types. The interview data were analyzed using thematic analysis [56]. An inductive approach was conducted through 6 phases [56] (see *Analysis of Teacher Interviews* in Multimedia Appendix 1) to gain insight into why teachers integrate PAL and PA breaks into academic lessons. In addition to the inductive approach, a deductive approach was used to identify teachers’ perceptions related to successful PAL adoption and implementation.

### Acute Effects of Physically Active Academic Lessons (Part 2)

#### Study Design and Participants

The effectiveness of physically active academic lessons among students was examined using a cluster randomized individual crossover trial conducted in two waves. In spring 2024, a total of 4 teachers of mathematics and foreign languages from 5 general upper secondary schools in Northern Ostrobothnia, who had participated in both the development project and the teacher interviews, were invited and agreed to implement the intervention. In autumn 2024, 2 schools from Central Finland were contacted, and a total of 5 teachers of mathematics and foreign languages from these schools expressed interest and agreed to implement the intervention. From a total of 17 eligible course groups of first- and second-year students, 168 students volunteered to participate in the study. These groups included 10 from schools in Northern Ostrobothnia (4 in mathematics and 6 in foreign languages), comprising 242 students, of whom 74 volunteered, and 7 from schools in Central Finland (4 in mathematics and 3 in foreign languages), comprising 200 students, of whom 94 volunteered. Students had the opportunity to withdraw from the study at any stage, and any data collected up to the point of withdrawal were retained as part of the research material. Eight students dropped out of the study.

**Figure 2.**
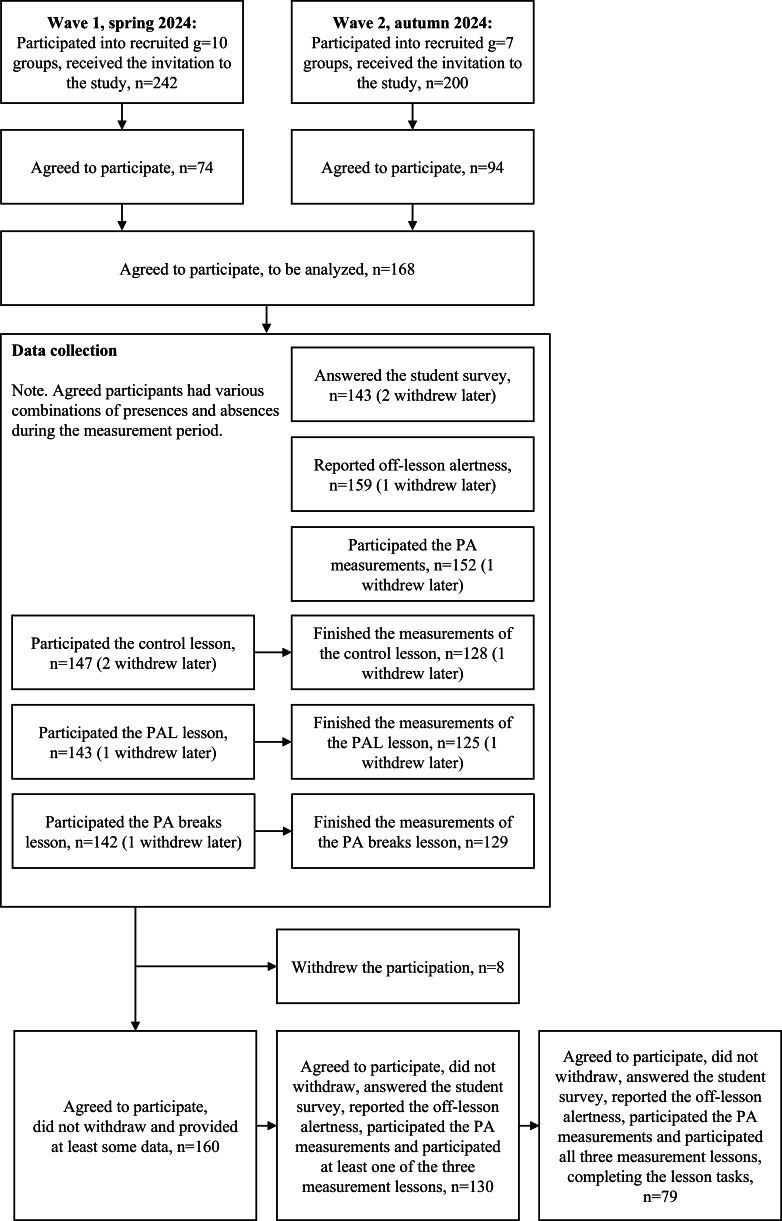
CONSORT flow diagram illustrating the study design of a cluster-randomized individual crossover trial among students. PA: physical activity; PAL: physically active learning.

Students in the same course group formed the clusters (mean cluster size 10, ranging from 1 to 18), which were randomized to undergo the 3 different treatments (lessons) in a different order. As there are 6 possible permutations of the 3 treatments, each cluster was allocated one of these sequences using a random number generator that sampled integers uniformly from 1 to 6. This procedure ensured randomized assignment of treatment order across the groups. After the trial, 30 voluntary students were invited to student interviews. This study followed the Consolidated Standards of Reporting Trials (CONSORT) statement (2025). The CONSORT flow diagram of the trial is presented in Figure 2.

#### Ethical Considerations

The study protocol was approved by the Ethics committee of Jamk University of Applied Sciences (Ethical review statement 472673, 21 December 2023). The study was prospectively registered (ISRCTN63809854, registered 11 January 2024 [57]).

Informed consent was obtained from the students and, in the case of minors, also from their guardians. This included consent for data collection as described in the information sheet and for the use and processing of personal data in accordance with the privacy notice. The consent form further included consent to being contacted after the trial for the purpose of inviting the students to a potential interview. Participation in the study was voluntary, and no financial or other compensation was offered for participation in the trial. Students who voluntarily chose to participate in the post‑trial interview were offered a €10 gift card (approximately US $11) as compensation.

With regard to privacy and confidentiality, all study data were pseudonymized prior to analysis, with personal identifiers replaced by ID numbers and accessible only to data managers/statisticians. All data are stored on secure, access‑controlled local servers at Jamk University of Applied Sciences with daily backups. Access is restricted to authorized research team members and managed by the principal investigator. No identifiable information will be reported.

#### Intervention

The first wave of the trial was delivered in spring 2024, and the second wave of the trial was delivered in autumn 2024. The trial focused on 2 subjects: mathematics and foreign languages, representing different types of subjects. Students participated in 3 different treatments (lessons) in random order. All lessons followed the National Core Curriculum for General Upper Secondary Education, but the teaching methods were different:

1. Treatment 1: Physically active mathematics or foreign language lessons with PAL (20 min PA is integrated into learning goals)
2. Treatment 2: Physically active mathematics or foreign language lessons with PA breaks (two 5-min breaks, including PA not related to learning goals)
3. Treatment 3: Typical, traditional math/foreign language lessons without PA (control lesson).

A description of the PAL methods and PA breaks used for treatment 1 and 2 is described in more detail (Textbox 1). The content of these methods was constructed in collaboration with general upper secondary school teachers, teacher educators, PA and learning researchers, and students.

Textbox 1. **Description of the content of the physically active learning (Treatment 1) and physical activity breaks used (Treatment 2).**

**Physically active learning**

Twenty minutes of PA (including instructions) were integrated into mathematics and foreign language lessons using station work. Station work was selected as the PAL method based on the results of a development project, “Moving academic lessons in general upper secondary school,” in which subject teachers identified it as a suitable approach across subjects. In this method, students solved subject-related tasks at different stations. The criteria for implementing station work were developed collaboratively by general upper secondary school teachers, teacher educators, and PA and learning researchers. These criteria included the number and location of stations, the type of PA integrated into tasks, and the transitions between stations.

Based on these criteria, 6-8 stations per lesson were set up in classrooms and/or other indoor spaces (eg, corridors), excluding outdoor areas. Transitions between stations could include climbing stairs, and students were instructed to walk briskly between stations. Approximately half of the station tasks involved PA, such as responding while moving. The physical activities were designed to be versatile and included balance, locomotor skills, manipulative skills, and strength (eg, squats, boxing, and precision throws). Most station tasks were completed in small groups, with some tasks performed in pairs.

Using these criteria, teachers planned lesson-specific tasks for each station with support from the research team. In addition to the station-based tasks, teachers planned the remainder of the lesson, which mainly consisted of teacher-led instruction, assigning and checking homework, and individual work using mathematics study books. The mathematics or foreign language tasks, including PA, were implemented in the middle of a 75-minute lesson, starting 25 minutes after the lesson began.

Teachers were instructed to rehearse the PAL (with different academic content) with the students to familiarize themselves and the students with the methods before the measurements.

**Physical activity breaks**

Two 3-minute PA breaks were added to mathematics and foreign language lessons. At the teachers’ request, prerecorded exercise videos were selected as the simplest format for implementing the PA breaks. Two different breaks were constructed from the Students on the Move program’s break exercise cards [58]. The breaks were planned to be versatile, including balance, locomotor skills, and strength exercises, and five cards per break were selected based on students’ preferences.

The PA breaks were implemented in the classroom, with the first break taking place 25 minutes after the lesson began and the second break 50 minutes after the lesson began (Break 1: [59]; Break 2: [60]). The breaks were mainly performed individually with the whole class participating, while the final exercises of each break were completed in pairs. The PA breaks did not include mathematics or foreign language learning goals, and teachers designed the lesson content apart from the breaks.

Before the measurements, teachers were instructed to rehearse the PA breaks with the students to familiarize both themselves and the students with the procedures.

Researchers held a 2-hour meeting to present the study, PAL method principles, and PA breaks to the teachers implementing the intervention. The PAL method (treatment 1) was similar for every group, but the lesson-specific content was designed by the teacher. This ensured that the lesson content was a part of the course learning objectives. Teachers had the opportunity to ask for assistance from research team members and were required to submit lesson plans. The PA breaks (treatment 2) were the same for every group, and teachers only needed to play them on the screen for the students. Because only some of the teachers and students were familiar with the different physically active classroom practices, the teachers were instructed to rehearse the PAL method and PA breaks with the students to familiarize themselves and the students with the methods before the measurements.

Given that the physically active lessons adhered to the national curriculum and consisted of tasks typical of students’ everyday lives, participation did not pose any risk beyond that of a regular school day. Despite the study’s hypotheses, PAL and PA breaks may cause negative effects, such as reduced situational engagement, alertness, or executive functions. These effects were measured and analyzed as part of the intervention outcomes, and subgroup analyses may reveal whether modifying factors are associated with perceived disadvantages.

The fidelity of the intervention was monitored by having each lesson observed by trained personnel, who recorded the course of the lesson and any deviations or unusual events. Apart from the addition of the second wave, no changes were made to the study protocol after trial registration, and no major deviations from the protocol occurred during the study.

#### Measurements

The majority of the measurements were performed as part of the lessons conducted by trained personnel. In the baseline measurement session, students’ executive functions (inhibition and working memory) and academic skills such as reading and math fluency and spatial visualization were measured. In addition, the devices (Axivity AX3 and Firstbeat Bodyguard 3 monitoring device) and instructions for measuring PA, sedentary time, stress, recovery, and sleep were distributed, and instructions for self-monitoring usual daily and hourly variations in alertness were given. Finally, personalized answer links to complete the background survey were sent to the students via email. Students completed the questionnaire in their free time.

Students wore the devices for two 4-to-5-day measurement periods (including the treatments). The first measurement period started straight after the baseline measurement session, or at the latest, in the morning 3 days before the first intervention lesson, and it ended in the morning after the first intervention lesson. The second measurement period started in the morning of the day before the second intervention lesson and ended in the morning after the last intervention lesson. Students also filled out an online alertness questionnaire 4 times per day (9 AM, 11 AM, 1 PM, and 3 PM) for 3 days. The timeline of the measurements is presented in Table 1.

**Table 1.** Example of a timetable of the measurements^a^.

	Day 1	Day 2	Day 3	Day 4	Day 5
Week 1	Baseline measurements. The first measurement period starts (devices on)	Alertness self-monitored at 9 AM, 11 AM, 1 PM, and 3 PM (devices on)	Alertness self-monitored at 9 AM, 11 AM, 1 PM, and 3 PM (devices on)	Intervention lesson 1 (devices on)	Devices removed
Week 2	Second measurement period starts (devices on). Alertness self-monitored at 9 AM, 11 AM, 1 PM, and 3 PM	Intervention lesson 2 (devices on)	Devices on	Intervention lesson 3 (devices on)	Devices removed

a For one group, the measurements lasted two weeks. During the first week of the study, the devices (Firstbeat Life and Axivity) were distributed during baseline measurements and were put on the same or the following day. The devices were worn for 3‐4 days continuously and were removed the day after the first intervention lesson. During the second week of the study, the devices were put on the day before the second intervention lesson and removed the day after the third intervention lesson. The measurement period in the second week lasted 2‐4 days continuously, depending on the group’s schedule.

**Figure 3.**
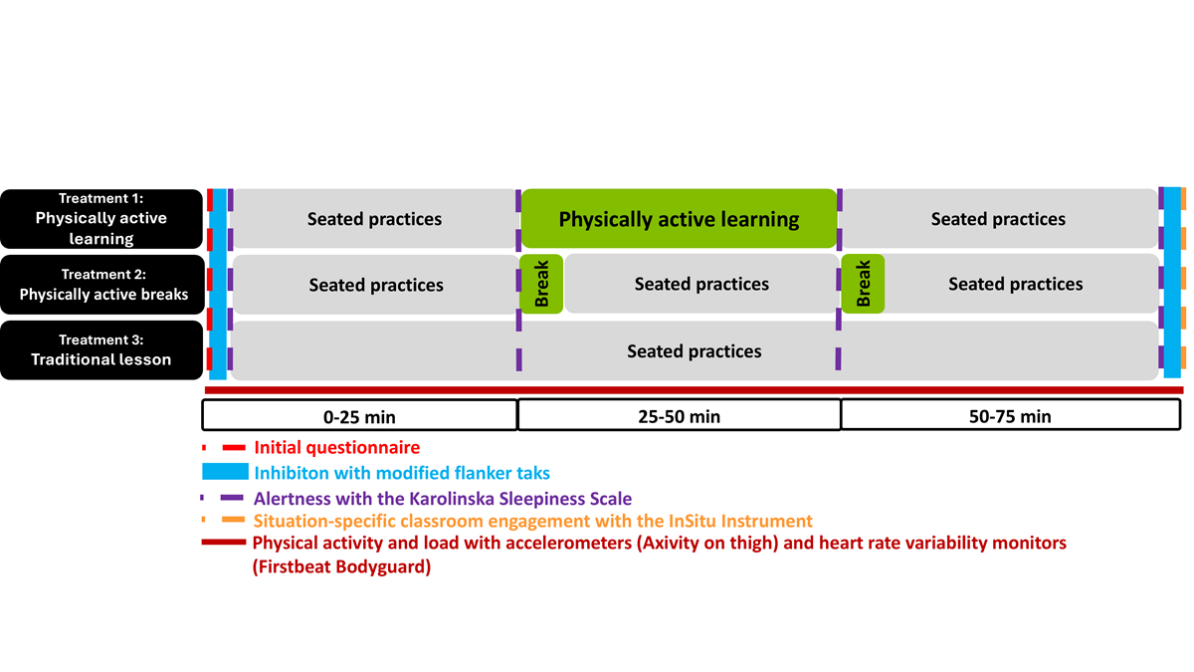
The structure of the intervention lessons. Measurements during the intervention lessons included a short initial survey, an executive function test, and an alertness questionnaire at the beginning of the lesson; two alertness questions during the lesson; and an alertness questionnaire, an executive function test, and a situational engagement questionnaire at the end of the lesson.

Measurements during the intervention lessons included a short initial survey, an executive function test, and an alertness questionnaire at the beginning of the lesson; 2 alertness questions during the lesson; and an alertness questionnaire, an executive function test, and a situational engagement questionnaire at the end of the lesson (Figure 3). The initial survey (2 minutes to complete), conducted on students' own computers through Visual Learning Environment (ViLLE), a widely used digital assessment and learning platform, included questions about the quality of the previous night's sleep, as well as the consumption of breakfast, lunch, caffeine, nicotine products, and PA before the treatment. Students also wore the Axivity AX3 and Firstbeat Bodyguard 3 monitors to assess PA, standing, sitting, stress, and recovery during the intervention lessons.

#### Main Outcomes

##### Alertness

Changes in alertness were assessed using the validated Karolinska Sleepiness Scale [61,62]. The Karolinska Sleepiness Scale is a single-item measure of subjective situational alertness on a 9-point scale (1=extremely alert, 9=extremely sleepy, fighting sleep). Students completed it on paper, and it took 0.5 minutes to complete.

##### Executive Function—Inhibition

Changes in executive function performance, particularly in inhibition, were measured during the treatments. Inhibition was assessed using a computerized, modified version of the Eriksen flanker task [63], in which participants completed up to 160 trials involving 5 horizontally aligned blue arrows presented in either congruous (the middle arrow facing the same direction as the flanking arrows) or incongruous (the middle arrow facing the opposite direction) conditions. Students were instructed to respond to the direction that the middle arrow was facing as quickly and accurately as possible. A fixed interstimulus interval of 1000 ms separated the trials. Accuracy (%) and reaction time for correct responses (ms) in congruent and incongruent trials were assessed. Students completed the flanker task on their own computers via the ViLLE platform, and it took 3 minutes. The task, although built on a different technology, mimics the NIH Toolbox version of the Flanker task, which has demonstrated developmental sensitivity and good test–retest reliability in both children and adults [64-66].

##### Situational Engagement

Situational engagement was assessed with the validated InSituations (InSitu) instrument [25]. The InSitu consists of 17 items rated on a 5-point scale (1=not at all; 5=very much) measuring (1) behavioral and cognitive engagement (7 items; eg, “How well did you concentrate during the lesson?”), (2) emotional engagement (3 items, eg, “How much did you like this lesson?”), (3) disaffection (3 items, eg, “How much did you do other things than the ongoing tasks and instruction?”), (4) competence experiences (2 items, eg, “How easy was the lesson for you?”), and (5) help seeking (2 items, eg, “How much did you ask for help from the teacher/another adult during the lesson?”). Students completed the InSitu on their own computers through the ViLLE platform, and it took 2 to 3 minutes to complete.

### Modifying Factors

#### Physical Load

To monitor physiological stress and recovery, students recorded a single-channel electrocardiogram to measure heart rate variability (HRV). HRV is measured as the time interval, in milliseconds, between two consecutive R-wave peaks (RR-Interval) in the electrocardiogram. The measurement was done using a Bodyguard 3 (Firstbeat Technologies Oy, Jyväskylä, Finland) and the Firstbeat Life app. The device was attached with self-adhesive single-use electrodes (Arbo H92SG). Participants wore the device around the clock, except for showering and water exercise. In the app, the students logged their activities in the supporting electronic diary.

Data were processed using Firstbeat research software. Firstbeat Life analysis is based on heart rate analysis software that identifies different physiological changes through HRV. Physiological recovery, stress, sleep, and PA were collected [67]. A score ranging from 0 to 100 was generated for each of the different physiological states for each day (morning to morning). A higher number represents a better score.

#### Mental Load

Mental load was assessed using questionnaires measuring study burnout and engagement as part of the background survey. The Student Burnout Inventory-9 [68] assesses study burnout through 9 items measuring exhaustion, cynicism, and inadequacy at school, rated on a 6-point scale (1=strongly disagree; 6=strongly agree). Meanwhile, Energy, Dedication, and Absorption-9 [69] assesses study engagement through 9 items, rated on a 7-point scale (0=not at all, 7=daily).

#### Physical Competence

Perceived physical competence was measured as part of the background survey using a questionnaire that included a modified Finnish version of the Perceived Physical Competence Scale for Children [70] and questions about the ability and desire to develop one’s own physical ability [71]. Altogether, they comprise 12 items rated on a 5-point scale.

#### Academic Competence

Perceived academic competence was assessed using a questionnaire including self-reported academic achievement score and self-perceptions (in math and foreign language) [72]. Perceived academic achievement score was assessed with a question “How would you rate your current academic performance in mathematics?*”* rated on a 4-point scale (1=excellent, 4=poor). Self-efficacy in math and foreign language was measured with 4 items (eg, “Mathematics/foreign language is an easy subject”) with a 5-point Likert scale (1=completely agree, 5=completely disagree).

### Other Study Variables

#### PA and Sedentary Time

To measure PA and sedentary time, students wore an accelerometer (AX3 [73]) on the anterior midline of either thigh. The accelerometer was attached to the skin using Opsite Flexifix transparent adhesive film (8 × 10 cm), with a piece of mesh between the monitor and skin to enhance breathability [74]. Envelopes including the monitor, detailed instructions including video link for how to attach the monitor, 5 pieces of adhesive film, and mesh swabs were delivered to participants face-to-face in a classroom. Data were collected with a 100 Hz sampling rate and a dynamic range of ±8 g.

Raw data were downloaded using OMGUI (version 1.0.0.45; Open Lab) [75]. ActiPASS (version 1.62) [76] was used to perform activity classification. Data were processed using the software’s default ProPASS settings, which have been validated to work well with children’s data as well as adults’ [77]. The start and end times, time at school, and intervention class times for each participant’s data were imported to ActiPASS using a diary file to inspect different time domains separately. The autocalibration function of the software was used. Activity was classified based on movement type: sleep, lie, sitting, standing, walking, running, stair-walking, cycling, but also based on the intensity of movement: sedentary, light, moderate, and vigorous PA.

#### Cognitive Functions and Academic Skills

To address baseline cognitive functions and academic skills, performance in the following tasks was evaluated through the ViLLE platform: the Choice Reaction Time task (as a warm-up task), the Sentence Reading Fluency task (assessing reading fluency), the Calculations task (assessing arithmetic fluency), the Digitized Corsi Blocks task (assessing working memory), the Spatial Relations subtask of the Woodcock-Johnson III Cognitive Assessment (assessing spatial visualization ability), and the Flanker task (assessing inhibition). Completing the tests took approximately 20 minutes. The tasks are described in more detail in Table S1 in Multimedia Appendix 1.

#### Background variables

The background survey was conducted electronically using Webropol software (Webropol Ltd, Helsinki, Finland), consisted of 55 questions and took a median time of 20 minutes to complete. The collected data included a comprehensive list of background variables: personal identifying information, age and sex, perceived health, height, weight, physical fitness, several questions on PA, sleep and wake-up times, nutrition, family form, parental socioeconomic questions, background academic performance in comprehensive school, and language syllabi in comprehensive school. Regarding the studies in upper secondary school, the questionnaire measured, for example, perceived performance and motivation in studies and diagnosed learning difficulties.

### Statistical Approaches

#### Analysis Plan

Generalized linear mixed-effects models will be applied to study the acute effects of the intervention on outcome measures. A cluster-randomized individual crossover design is assumed, that is, individuals serve as their own controls when the clusters (school classes) are crossed over the three intervention arms. This is the most efficient of cluster-randomized study designs [78]. The data will be analyzed according to intention-to-treat principles. Data lost due to participation withdrawals, student absences from measurement lessons, and missingness caused by other reasons will be multiply imputed in the analysis of primary outcome variables.

Within the analysis of main outcomes of classroom interventions (alertness, executive function, and situational engagement), the first analyses will quantify the main intervention effects on the outcomes. The main effects in generalized linear mixed-effects models will be adjusted for 3 levels of confounding factors. First, the factors that are consequences of the study design are taken into account (season, lesson time, subject, and group ID as a random intercept). Second, the student-level constant factors (sex, learning-related disabilities) will be included in the models. Finally, the third set of models will be additionally adjusted for individual lesson-specific factors (prelesson activities, baseline alertness, quality and duration of preceding sleep, preceding meals, smoking and caffeine intake, and menstruation status). The further manuscripts will study modifying factors for the intervention effects. The studied modifying factors are planned to include current physical and mental load, perceived physical competence, and perceived academic competence. The models will include corresponding or less extensive adjustments based on the observations of analysis of main intervention effects. Mplus and R statistical packages will be used.

#### Sample Size Determination

Prerecruitment power calculations were conducted to determine the required sample size for the study. Required statistical power (1-β) was set to 90%, and significance level (α) was set at .05. The study was planned to detect medium intervention main effects (Cohen *d*=0.50) in the primary outcome variables: situational engagement, alertness, and inhibition. Definition of the desired minimum detectable effect size was, as usual, a compromise between academic desire and study costs and supported by published works on the outcomes (eg, alertness [79,80], inhibition [81,82]).

The required sample size *n* for an individually randomized controlled trial would be

$n_{IRCT}=\frac{{\left(Z_{1-\alpha /2}+Z_{1-\beta }\right)}^{2}\cdot 2\sigma^{2}}{\Delta^{2}}$,

where $Z_{x}$ is the *x*th quantile of the standard normal distribution, α is the size of the test (ie, level of significance), β is 1 – required level of statistical power, Δ is the minimum detected difference in the levels of outcome, and $\sigma^{2}$ is the assumed overall variance of the outcome variable.

In educational interventions, participants are most often divided into school classes, and therefore, individual randomization for intervention groups is rarely feasible. This cluster-randomization weakens the design generally, and the required sample size increases by a factor called the design effect.

In our study design, individuals serve as their own controls by visiting the two intervention treatments and a control treatment in randomized order. The design effect gets a form of

$DE_{CRIC}=2\left(1-\rho_{1}-\rho_{2}\right)$,

where $\rho_{1}$ is the assumed intra-subject correlation and $\rho_{2}$ is the assumed intra-cluster correlation.

Assuming the variance structure as observed in the pilot study (Matikkavire), $\rho_{1}=0.31$ and $\rho_{2}=0.01,$ the design effect is $2\left(1-0.31-0.01\right)=1.36$ and the required sample is n=115 individuals to be crossed over the treatments. By assuming 33 students per class and a 70% student participation rate, we need to recruit five classes. To balance the design, we decided to recruit six classes such that two classes would have been randomized to each of the three intervention arms, where the order of treatments is rotated. As the number of volunteering participants per group remained lower than expected, more groups were invited, and eventually, the recruitment continued in a second wave in the autumn of 2024 to obtain the desired statistical power.

The forthcoming subgroup analyses (eg, sex-specific analyses) and moderation analyses (moderating factors will include physiological and psychological state, and physical and academic competence of the student) have less statistical power to detect medium interaction effects. Statistical power is approximated for subgroups by standard methods as discussed above, and for more complex moderation analyses by, eg, post data-collection Monte Carlo simulation. The post hoc power calculations are not used to determine sample size, but to support the confidence of the interpretation of the results of the moderation models.

### Student interviews (Part 3)

The evaluation of students’ experiences and perceptions of physically active academic lessons in a general upper secondary school will be conducted in the context of the cluster-randomized individual crossover trial described above. Of the 74 students recruited in the first wave of the trial, 59 consented (21 from mathematics lessons and 38 from foreign language lessons) to being invited for an interview (Figure 1). The aim is to interview as wide a range of students as possible; therefore, students will be selected in different ways. While one subject teacher proposed potential interviewees to the researcher, most students expressed their willingness to participate during their initial face-to-face contact with the researcher following the intervention. Thirty students were contacted and agreed to participate in the interviews in spring 2024. Ten students participated in the trial during mathematics lessons (5 taking the short syllabus and 5 the long syllabus), and 20 students during foreign language lessons (8 in English, 13 in Swedish, and 1 in German).

All 30 students were interviewed after participating in all three different lessons (treatments; see Figure 3). Ten students will be interviewed virtually via Microsoft Teams, and 20 students will be interviewed in person at their schools using an Olympus LS-PS-1 audio recorder. Semistructured interviews were chosen as a method to enable open and spontaneous responses and to capture the essence of students’ experience [83]. The questions are organized around the main themes (see Student interviews in the Supplemental material in Multimedia Appendix 1). The duration of the interviews will range from 37 to 70 minutes. The student responses will be transcribed and analyzed by using the engagement framework [23] and thematic analysis [56]. Thematic analysis is suitable for identifying patterns within and across data in relation to participants’ lived experience, views, and perspectives when the study aims to understand what participants think, feel, and do. Both deductive (literature-driven dimensions) and inductive (emerging from participants’ data) approaches will be used. The main focus of using the situational engagement framework is to analyze students’ responses to PAL. In addition, responses to PA breaks allow for a comparison of how PAL and PA breaks influence situational engagement.

## Results

Data collection has been completed. Fourteen teachers participated in the interviews (Part 1), 168 students participated in a cluster-randomized individual crossover trial in mathematics and foreign language lessons (Part 2), and 30 students participated in post-trial interviews (Part 3). The participating students received personalized feedback on their PA, physiological recovery, stress, and sleep measurements. In addition, teachers who conducted the intervention lessons received school-specific summaries, including information on students’ levels of PA, sleep patterns, and engagement during lessons. The results of the study will be reported in separate manuscripts, and the first findings based on the data analysis are expected by spring 2026. The results will be disseminated through plain-language summaries tailored for teachers and schools, published reports in the trial registry, and presentations at scientific and national seminars aimed at researchers and practitioners.

## Discussion

### Principal Results

This multidisciplinary mixed methods study aims to evaluate the experiences and perceptions of both teachers and students during physically active academic lessons in a general upper secondary school. The research interest is especially in facilitators, barriers, usefulness, and meaning of physically active academic lessons for the situational learning process. Additionally, the purpose is to examine the immediate effects of PAL and PA breaks on cognitive prerequisites for learning such as alertness and executive functions, and situational engagement, including behavioral, emotional, and cognitive engagement, as well as disaffection, competence experiences, and help-seeking among secondary school students. The study also aims to explore factors that modify these effects, such as physical and mental load, as well as perceived physical and academic competence. Aligning with previous findings, we hypothesize that this study will confirm the benefits [26-29], feasibility, and usability [41,43,50,51] of physically active classroom practices in general upper secondary education. Moreover, we expect the study to provide new insights into the role of PAL and PA breaks in situational learning processes in authentic educational settings, considering both teacher and student perspectives as well as potential moderating factors.

### Comparison With Prior Work

This is one of the first studies to explore experiences related to teaching, learning processes, and optimal instructions during physically active academic lessons in general upper secondary schools, while also examining the immediate effects of PAL and PA breaks on situational engagement and cognitive prerequisites for learning. Furthermore, the strength of this study lies in its combination of solid knowledge in both teaching and PA, allowing it to examine the phenomenon from both the student’s and the subject teacher’s perspectives at different levels of action (collected experiences and measured prerequisites for learning). A further strength of the study is that PA was measured from the thigh, enabling the distinction between sitting and standing during lessons. Additionally, HRV was monitored online to assess the balance between stress and recovery during classroom activities, which adds a novel dimension to the research.

### Implications and Future Research

The study’s findings will offer crucial, evidence-based insights into active classroom practices, aiding subject teachers and teacher education programs. This support helps teachers and schools adopt effective and innovative instructional strategies, pedagogical models, and designs, ultimately enhancing student learning and well-being in upper secondary education. Future research should study the phenomenon in other educational settings for older students, such as vocational secondary education and higher education, and across different cultural contexts. Research on the acute effects and students’ experiences should be extended to other subjects beyond mathematics and languages.

### Limitations

The study has faced certain limitations. The participation rate in the study was lower than desired, and possibly, less active or academically weaker students did not participate in the study, which may weaken the generalizability of the study results. The generalizability of teachers’ experiences is limited, as the findings are based on fourteen interviews from one municipality. While the interviews offer varied insights into integrating PA into different subjects, the experiences of individual subjects often rely on just one or two teachers. The teachers who participated were interested in the topic, but the views of less interested teachers were not captured. However, a couple of teachers who withdrew from the development project were also interviewed, which helps to understand why implementing PAL and PA breaks can be challenging.

### Conclusions

This study aims to address a gap in the literature by evaluating the feasibility and effectiveness of integrating physically active methods into academic lessons in upper secondary schools. Using a mixed-methods cluster-randomized individual crossover trial, the study seeks to enhance students’ learning and well-being.

## Supplementary material

10.2196/84601 Multimedia Appendix 1 Supplemental material on teacher and student interviews and measurements of cognitive and academic skills.

10.2196/84601 Checklist 1 SPIRIT 2025 checklist.

## Abbreviations

CONSORT: Consolidated Standards of Reporting Trials
HRV: heart rate variability
InSitu: InSituations
PA: physical activity
PAAL: Physically Active Academic Lessons
PAL: physically active learning
SPIRIT: Standard Protocol Items: Recommendations for Interventional Trials
ViLLE: Visual Learning Environment

## References

[1] (2017). PISA 2015 Results (Volume III) Students’ Well-Being.

[2] (2009). Global health risks: mortality and burden of disease attributable to selected major risks. https://iris.who.int/server/api/core/bitstreams/50e6ba96-c5c3-4e1d-b635-f111bb74f4bf/content.

[3] Twenge JM, Cooper AB, Joiner TE, Duffy ME, Binau SG (2019). Age, period, and cohort trends in mood disorder indicators and suicide-related outcomes in a nationally representative dataset, 2005-2017. J Abnorm Psychol.

[4] (2022). Student barometer in general upper secondary school [Article in Finnish]. The Finnish Upper Secondary Students’ Union.

[5] Salmela-Aro K, Savolainen H, Holopainen L (2009). Depressive symptoms and school burnout during adolescence: evidence from two cross-lagged longitudinal Studies. J Youth Adolescence.

[6] Madigan DJ, Curran T (2021). Does burnout affect academic achievement? A meta-analysis of over 100,000 students. Educ Psychol Rev.

[7] Guthold R, Stevens GA, Riley LM, Bull FC (2020). Global trends in insufficient physical activity among adolescents: a pooled analysis of 298 population-based surveys with 1·6 million participants. Lancet Child Adolesc Health.

[8] (2022). Finland’s report card 2022 on physical activity for children and youth. 2022. LIKES research reports on physical activity and health 407. https://www.activehealthykids.org/wp-content/uploads/2022/03/Finland-report-card-long-form-2022.pdf.

[9] Chaput JP, Willumsen J, Bull F (2020). 2020 WHO guidelines on physical activity and sedentary behaviour for children and adolescents aged 5-17 years: summary of the evidence. Int J Behav Nutr Phys Act.

[10] Norris E, van Steen T, Direito A, Stamatakis E (2020). Physically active lessons in schools and their impact on physical activity, educational, health and cognition outcomes: a systematic review and meta-analysis. Br J Sports Med.

[11] Bedard C, St John L, Bremer E, Graham JD, Cairney J (2018). A systematic review and meta-analysis on the effects of physically active classrooms on educational and enjoyment outcomes in school age children. PLoS ONE.

[12] Watson A, Timperio A, Brown H, Best K, Hesketh KD (2017). Effect of classroom-based physical activity interventions on academic and physical activity outcomes: a systematic review and meta-analysis. Int J Behav Nutr Phys Act.

[13] Sneck S, Viholainen H, Syväoja H (2019). Effects of school-based physical activity on mathematics performance in children: a systematic review. Int J Behav Nutr Phys Act.

[14] Daly-Smith AJ, Zwolinsky S, McKenna J, Tomporowski PD, Defeyter MA, Manley A (2018). Systematic review of acute physically active learning and classroom movement breaks on children’s physical activity, cognition, academic performance and classroom behaviour: understanding critical design features. BMJ Open Sport Exerc Med.

[15] Masini A, Marini S, Gori D, Leoni E, Rochira A, Dallolio L (2020). Evaluation of school-based interventions of active breaks in primary schools: a systematic review and meta-analysis. J Sci Med Sport.

[16] de Greeff JW, Bosker RJ, Oosterlaan J, Visscher C, Hartman E (2018). Effects of physical activity on executive functions, attention and academic performance in preadolescent children: a meta-analysis. J Sci Med Sport.

[17] Diamond A (2013). Executive functions. Annu Rev Psychol.

[18] Best JR, Miller PH, Naglieri JA (2011). Relations between executive function and academic achievement from ages 5 to 17 in a large, representative national sample. Learn Individ Differ.

[19] Mavilidi MF, Ruiter M, Schmidt M (2018). A narrative review of school-based physical activity for enhancing cognition and learning: the importance of relevancy and integration. Front Psychol.

[20] Bartholomew JB, Jowers EM (2011). Physically active academic lessons in elementary children. Prev Med.

[21] Daly-Smith A, Quarmby T, Archbold VSJ (2020). Implementing physically active learning: Future directions for research, policy, and practice. J Sport Health Sci.

[22] Fredricks JA, Blumenfeld PC, Paris AH (2004). School engagement: potential of the concept, state of the evidence. Rev Educ Res.

[23] Fredricks JA, Filsecker M, Lawson MA (2016). Student engagement, context, and adjustment: addressing definitional, measurement, and methodological issues. Learn Instr.

[24] Xerri MJ, Radford K, Shacklock K (2018). Student engagement in academic activities: a social support perspective. High Educ.

[25] Vasalampi K, Muotka J, Pöysä S, Lerkkanen MK, Poikkeus AM, Nurmi JE (2016). Assessment of students’ situation-specific classroom engagement by an InSitu Instrument. Learn Individ Differ.

[26] Haverkamp BF, Wiersma R, Vertessen K, van Ewijk H, Oosterlaan J, Hartman E (2020). Effects of physical activity interventions on cognitive outcomes and academic performance in adolescents and young adults: a meta-analysis. J Sports Sci.

[27] Verburgh L, Königs M, Scherder EJA, Oosterlaan J (2014). Physical exercise and executive functions in preadolescent children, adolescents and young adults: a meta-analysis. Br J Sports Med.

[28] Mavilidi MF, Mason C, Leahy AA (2021). Effect of a time-efficient physical activity intervention on senior school students’ on-task behaviour and subjective vitality: the ‘burn 2 learn’ cluster randomised controlled trial. Educ Psychol Rev.

[29] Robinson KJ, Lubans DR, Mavilidi MF (2022). Effects of classroom-based resistance training with and without cognitive training on adolescents’ cognitive function, on-task behavior, and muscular fitness. Front Psychol.

[30] Donnelly JE, Hillman CH, Castelli D (2016). Physical activity, fitness, cognitive function, and academic achievement in children: a systematic review. Med Sci Sports Exerc.

[31] Singh AS, Saliasi E, van den Berg V (2019). Effects of physical activity interventions on cognitive and academic performance in children and adolescents: a novel combination of a systematic review and recommendations from an expert panel. Br J Sports Med.

[32] Jafflin K, Pfeiffer C, Bergman MM (2019). Effects of self-esteem and stress on self-assessed health: a Swiss study from adolescence to early adulthood. Qual Life Res.

[33] Matud MP, Díaz A, Bethencourt JM, Ibáñez I (2020). Stress and p[sychological distress in emerging adulthood: a gender analysis. J Clin Med.

[34] Girotti M, Adler SM, Bulin SE, Fucich EA, Paredes D, Morilak DA (2018). Prefrontal cortex executive processes affected by stress in health and disease. Prog Neuropsychopharmacol Biol Psychiatry.

[35] Dong Z, Wang P, Xin X (2022). The relationship between physical activity and trait anxiety in college students: the mediating role of executive function. Front Hum Neurosci.

[36] Stults-Kolehmainen MA, Sinha R (2014). The effects of stress on physical activity and exercise. Sports Med.

[37] Timo J, Sami YP, Anthony W, Jarmo L (2016). Perceived physical competence towards physical activity, and motivation and enjoyment in physical education as longitudinal predictors of adolescents’ self-reported physical activity. J Sci Med Sport.

[38] Lindner KJ (2002). The physical activity participation–academic performance relationship revisited: perceived and actual performance and the effect of banding (academic tracking). Pediatr Exerc Sci.

[39] Ferla J, Valcke M, Schuyten G (2010). Judgments of self-perceived academic competence and their differential impact on students’ achievement motivation, learning approach, and academic performance. Eur J Psychol Educ.

[40] Sneck S, Syväoja H, Järvelä S, Tammelin T (2023). More active lessons: teachers’ perceptions of student engagement during physically active maths lessons in Finland. Education Inquiry.

[41] Schmidt SK, Bratland-Sanda S, Bongaardt R (2022). Secondary school teachers’ experiences with classroom-based physically active learning: “I’m excited, but it’s really hard”. Teach Teach Educ.

[42] Teslo S, Thurston M, Lerum Ø (2023). Teachers’ sensemaking of physically active learning: a qualitative study of primary and secondary school teachers participating in a continuing professional development program in Norway. Teach Teach Educ.

[43] Lerum Ø, Bartholomew J, McKay H (2019). Active smarter teachers: primary school teachers’ perceptions and maintenance of a school-based physical activity intervention. Transl J ACSM.

[44] Pöysä S, Vasalampi K, Muotka J, Lerkkanen MK, Poikkeus AM, Nurmi JE (2018). Variation in situation-specific engagement among lower secondary school students. Learn Instr.

[45] Fröberg A, Jonsson L, Berg C (2018). Effects of an empowerment-based health-promotion school intervention on physical activity and sedentary time among adolescents in a multicultural area. Int J Environ Res Public Health.

[46] Mullins NM, Michaliszyn SF, Kelly-Miller N, Groll L (2019). Elementary school classroom physical activity breaks: student, teacher, and facilitator perspectives. Adv Physiol Educ.

[47] Howie EK, Newman-Norlund RD, Pate RR (2014). Smiles count but minutes matter: responses to classroom exercise breaks. Am J Health Behav.

[48] Sneck S, Järvelä S, Syväoja H, Tammelin T (2022). Pupils’ experiences and perceptions of engagement during the Moving Maths programme. Educ 3 13.

[49] Riley N, Lubans D, Holmes K, Hansen V, Gore J, Morgan P (2017). Movement-based mathematics: enjoyment and engagement without compromising learning through the EASY minds program. EURASIA J MATH SCI T.

[50] Fred N, Mikkonen K, Pramila-Savukoski S, Hylkilä K, Kuivila HM (2023). Upper secondary school students’ experiences of how exercise breaks affect their well-being and ability to study: a qualitative study. Int J Res Educ Sci.

[51] Romar JE, Enlund M, Lind S, Björkgren M (2023). Movement integration in academic classrooms; a focus on secondary students’ experiences. J Phys Educ Sport.

[52] Mulhearn SC, Kulinna PH, Webster C (2020). Stakeholders’ perceptions of implementation of a comprehensive school physical activity program: a review. Kinesiol Rev (Champaign).

[53] Marttinen RHJ, McLoughlin G, Fredrick R, Novak D (2017). Integration and physical education: a review of research. Quest.

[54] (2019). The ethical principles of research with human participants and ethical review in the human sciences in Finland. https://tenk.fi/sites/default/files/2021-01/Ethical_review_in_human_sciences_2020.pdf.

[55] Daly-Smith A, Morris JL, Norris E (2021). Behaviours that prompt primary school teachers to adopt and implement physically active learning: a meta synthesis of qualitative evidence. Int J Behav Nutr Phys Act.

[56] Braun V, Clarke V (2006). Using thematic analysis in psychology. Qual Res Psychol.

[57] Physically active academic lessons in general upper secondary school - student study. ISRCTN.

[58] Liikkuva opiskelu. https://liikkuvaopiskelu.fi/tukimateriaali/tulostettavat-taukoliikuntakortit/.

[59] Taukoliikuntavideoita fyysisesti aktiivisiin oppitunteihin 1. https://www.youtube.com/watch?v=85R7H8-WFZo.

[60] Taukoliikuntavideoita fyysisesti aktiivisiin oppitunteihin 2. https://www.youtube.com/watch?v=YfX_4p3VIGY.

[61] Akerstedt T, Gillberg M (1990). Subjective and objective sleepiness in the active individual. Int J Neurosci.

[62] Shochat T, Santhi N, Herer P, Dijk DJ, Skeldon AC (2021). Sleepiness is a signal to go to bed: data and model simulations. Sleep.

[63] Eriksen BA, Eriksen CW (1974). Effects of noise letters upon the identification of a target letter in a nonsearch task. Percept Psychophys.

[64] Zelazo PD, Anderson JE, Richler J (2014). NIH toolbox cognition battery (CB): validation of executive function measures in adults. J Int Neuropsychol Soc.

[65] Zelazo PD, Anderson JE, Richler J, Wallner-Allen K, Beaumont JL, Weintraub S (2013). II. NIH toolbox cognition battery (CB): measuring executive function and attention. Monogr Soc Res Child Dev.

[66] Weintraub S, Dikmen SS, Heaton RK (2013). Cognition assessment using the NIH Toolbox. Neurology (ECronicon).

[67] (2014). Stress and recovery analysis method based on 24-hour heart rate variability. https://assets.firstbeat.com/firstbeat/uploads/2015/10/Stress-and-recovery_white-paper_20145.pdf.

[68] Salmela-Aro K, Kiuru N, Leskinen E, Nurmi JE (2009). School burnout inventory (SBI). Eur J Psychol Assess.

[69] Salmela-Aro K, Upadaya K (2012). The schoolwork engagement inventory. Eur J Psychol Assess.

[70] Lintunen T (1987). Perceived physical competence scale for children. Scand J Sport Sci.

[71] Hirvensalo M, Jaakkola T, Sääkslahti A, Lintunen T (2016). Lasten Ja Nuorten Liikuntakäyttäytyminen Suomessa LIITU-Tutkimuksen Tuloksia 2016.

[72] Metsämuuronen J (2012). Challenges of the Fennema-Sherman test in the international comparisons. IJPS.

[73] Axivity. https://axivity.com/product/ax3.

[74] Schneller MB, Bentsen P, Nielsen G (2017). Measuring children’s physical activity: compliance using skin-taped accelerometers. Med Sci Sports Exerc.

[75] Jackson D (2024). OMGUI (version 1.0.0.45). Github.

[76] Hettiarachchi P, Johansson P (2025). ActiPASS (version 2025.04). Zenodo.

[77] Lendt C, Hettiarachchi P, Johansson PJ (2024). Assessing the accuracy of activity classification using thigh-worn accelerometry: a validation study of ActiPASS in school-aged children. J Phys Act Health.

[78] Rutterford C, Copas A, Eldridge S (2015). Methods for sample size determination in cluster randomized trials. Int J Epidemiol.

[79] Campbell IG, Burright CS, Kraus AM, Grimm KJ, Feinberg I (2017). Daytime sleepiness increases with age in early adolescence: a sleep restriction dose-response study. Sleep.

[80] Sallinen M, Härmä M, Akila R (2004). The effects of sleep debt and monotonous work on sleepiness and performance during a 12-h dayshift. J Sleep Res.

[81] Hillman CH, Pontifex MB, Raine LB, Castelli DM, Hall EE, Kramer AF (2009). The effect of acute treadmill walking on cognitive control and academic achievement in preadolescent children. Neuroscience.

[82] Stroth S, Kubesch S, Dieterle K, Ruchsow M, Heim R, Kiefer M (2009). Physical fitness, but not acute exercise modulates event-related potential indices for executive control in healthy adolescents. Brain Res.

[83] Hirsjärvi S, Hurme H (2000). Tutkimushaastattelu: Teemahaastattelun Teoria Ja Käytäntö.

