# Supplementary material for: Enhancing Upper Secondary Students’ Situational Engagement and Cognitive Prerequisites of Learning Through the Physically Active Academic Lessons Intervention: Protocol for a Mixed Methods Cluster Randomized Individual Crossover Trial
Source: JMIR Res Protoc. 2026 Feb 3;15:e84601. doi: 10.2196/84601 (PMC12867474; doi:10.2196/84601)
Supplement: Multimedia Appendix 1 [file resprot-v15-e84601-s001.pdf]

Multimedia Appendix 1

Teacher interviews

Main themes of the subject teacher interviews are presented below.

BACKGROUND QUESTIONS

Age
Gender
Teaching subjects
Teaching experience

THE PROCESS OF ADOPTING AND IMPLEMENTING PHYSICALLY ACTIVE LEARNING (PAL) AND PHYSICAL ACTIVITY (PA) BREAKS

How would you describe your development and journey toward becoming a teacher who implements physically active lessons?
Teachers' beliefs about their own capabilities
Teacher training

PAL

1) BENEFITS AND DRAWBACKS

Which physically active classroom practice do you prefer to implement in your teaching, and why?

Think of a lesson where you successfully implemented PAL. What happened in the classroom during and after the activity? What factors contributed to the success of the activity?

Think of a lesson where implementing PAL didn't go as well as you had hoped. What happened in the classroom during and after the activity? Why do you think it didn't work as well?

What experiences have you had with PAL in relation to the learning goals of your subject?

How would you describe the teacher's role during PAL?
How would you describe the students' role during PAL?

2) DELIVERY

What kinds of experiences have you had implementing PAL in different grades, with different courses in your subject, in various phases of the lesson, at different points in the academic year, and in various stages of the learning process (during learning, practice, and repetition)?

How would you describe the intensity and frequency of PAL implementation?

Based on your experience, what types of physically active classroom practices work in your subject area at the upper secondary level?
What types of physically active classroom practices do not work well in your subject area at the upper secondary level?

3) RESOURCES

In which learning environments do you implement physically active methods, and why do you choose these environments?

4) WHOLE SCHOOL APPROACH

What factors facilitate the implementation of physically active methods?
What factors prevent the implementation of physically active methods?

5) STUDENTS' PERCEPTIONS OF PAL

What is your perception of how students experience PAL?

PA BREAKS

1) BENEFITS AND DRAWBACKS

Which types of PA breaks do you prefer to implement during your lessons, and when and why?

Think of lessons where you have successfully implemented PA breaks. What happened in the classroom during and after the PA break? What factors contributed to the success of the break(s)?

Think of a lesson where implementing a PA break didn't go as well as you had hoped. What happened in the classroom during and after the PA break? Why didn't the PA break work well?

Based on your experience, what types of PA breaks work in the upper secondary level?
What types of PA breaks do not work in in upper secondary level?

2) DELIVERY

How would you describe the intensity and frequency of implementing PA breaks?

What kinds of experiences have you had implementing PA breaks in various phases of the lesson?

Based on your experience, what types of PA breaks work at the upper secondary level?
What types of PA breaks do not work at the upper secondary level?

What facilitates the implementation of PA breaks?
What prevents the implementation of PA breaks?

3) STUDENTS' PERCEPTIONS OF PA BREAKS

What is your perception of how students experience PA breaks?

Analysis of teacher interviews

The thematic analysis process began with transcribing the interviews, followed by repeated reading to achieve familiarization with the entire data set. Inductive approach is conducted through six phases[1] to gain insight into why teachers integrate PAL and PA breaks into academic lessons: 1. familiarization with the data through reading and noting initial ideas for coding; 2. coding by writing codes directly on the text and compiling a list of codes; 3. searching for themes by organizing coded pieces of paper into initial themes, exploring relationships between codes using thematic maps (visual representations), and developing preliminary themes that included sub-themes and all coded data extracts; 4. reviewing themes by re-reading the entire data set and checking that themes coherently represent the data; 5. clarifying why extracts are analytically relevant, and defining sub-themes and themes by assigning names that clearly describe them; 6. writing up the report, including presenting a valid and nuanced account of the data within and across themes, supported by vivid data extracts. A deductive approach is used to identify teachers' perceptions related to successful PAL adoption and implementation.

1) Braun V, Clarke V. Using thematic analysis in psychology. Qual. Res. Psychol;2006;3(2):77–101. doi:10.1191/1478088706qp063oa

Methods for measuring cognitive functions and academic skills in a cluster-randomized individual crossover trial

Table S1. A detailed description of the tasks measuring baseline cognitive functions and academic skills.

	Measuring	Execution	Items	Determination	Result	Notice
Choice reaction time task	Warm-up task	Participants are presented with two squares, colored red or black. Participants must decide as quickly as possible whether the colors are the same or different.	16 items, fixed inter-stimulus interval of 1000 ms, response time limit of 5000 ms.		Number of correct responses.	
Sentence Reading Fluency	Reading fluency	Participants are presented with a series of simple written sentences and must quickly determine whether each sentence is true or false (e.g., “Apples grow on trees”).	Sentences are designed to be easily decodable and focus on general knowledge or common-sense content.	Participants have 90 seconds to complete as many items as possible.	Number of correct answers.	Standardized for 3rd to 9th graders, part of the FUNA reading assessment battery
Calculations	Arithmetic fluency	Participants are presented with calculation tasks (+, –, ×, ÷) in increasing difficulty. The subject is asked to solve as many calculations correctly as possible within the time limit.		Maximum total time is 5 minutes; maximum time per item is 60 seconds. If the subject answers incorrectly in 5 out of 6 consecutive items, the task ends early.	Number of correct answers	Standardized for 3rd to 9th graders, part of the FUNA math assessment battery

Digitized Corsi Blocks	Working memory	Participants are presented with a 5×5 grid of squares. Starting with 2 squares, a sequence flashes. After the sequence, participants must click the same squares in the same order.	Delay between items is 1500 ms; flash speed is 750 ms with no delay between flashes.	Four attempts per sequence length. The sequence length increases by one if the participant gets 3 out of 4 correct.	Highest correctly repeated sequence length.	The number of flashes and feedback is displayed on the screen.
Woodcock-Johnson -III Cognitive Assessment – Spatial Relations	Spatial visualization ability	Participants are shown a target shape and must select the correct combination of pieces that can be mentally rotated and assembled to match the target. No physical manipulation is required. Participants must identify the correct answer from multiple-choice options based on visual inspection.	Task difficulty increases progressively with more complex shapes and distractors.	Participants have 3 minutes to solve as many items as possible. The task ends after 3 consecutive errors. Total of 31 items.	Number of correct answers.	
Flanker task	Inhibition	Participants are shown blue arrows in either congruent (middle arrow faces the same direction as flanking arrows) or incongruent (middle arrow faces opposite direction) conditions. They must respond to the direction of the middle arrow as quickly and accurately as possible.	160 trials with five horizontally aligned blue arrows. Fixed inter-stimulus interval (ISI) of 1000 ms between trials.	All trials were completed within a 3-minute time limit.	Reaction time and number of correct responses for both congruent and incongruent trials.	

Student interviews

Main themes of the student interviews are presented below.

BACKGROUND QUESTIONS

Year course
Name of the course
Feelings about general upper secondary school studies in general
Opinion about the peaceful studying environment
Definition of physically active academic learning

PHYSICALLY ACTIVE LEARNING (PAL):

1) STUDENTS' PERCEPTIONS OF PAL

Was this your first time when you took part in physically active learning?
What did you think about the station work?

How much did you enjoy studying like this?

How did physically active tasks contribute to your learning during this lesson? Describe closely what you mean.

How do you perceive the importance of making lessons physically more active in general upper secondary school?

Do you feel that learning in a physically active way has any drawbacks to your learning?

What kind of students benefit the most if lessons include physically active learning methods?

In your opinion, which subjects are suitable for PAL?

Have PAL methods changed your thinking about learning? Motivate your answer.

2) EXPERIENCES

What kind of experiences have you had with PAL? What kinds of activities have you participated in? Give an example that you remember particularly well.

What do you consider to be a good and useful PAL task? What kind of PAL tasks have you enjoyed the most? Based on your experience, what are effective ways to implement PAL in academic subjects?
How do you feel about the PAL in academic lessons? What kind of PAL tasks do you like or dislike? Why?

How does PAL affect you? How does active learning affect your alertness and concentration?

Based on your experience, what are effective ways to implement PAL in academic lessons?

What are the benefits of PAL?

What factors prevent learning in lessons that include PAL methods?

Do you think that students become tired of learning in a physically active way?

3) LEARNING ENVIRONMENT

How often would you like to have PAL tasks? How much physically active learning should there be in a general upper secondary school, in general, or in this subject during one period?

Where do you prefer studying in a physically active way? (Inside? In the classroom? In the hallway? Elsewhere in the school building? Outside?) Why?

PHYSICAL ACTIVITY (PA) BREAKS:

1) STUDENTS' PERCEPTIONS OF PA BREAKS

What do you think of the PA breaks?

Do you think that PA breaks negatively affect you and your learning? Motivate your answer.

2) PARTICIPATION AND EXPERIENCES

Do you always participate in PA breaks? What motivates you most to participate in PA breaks? If you haven't participated in the PA breaks, why do you prefer to sit?

Which types of PA breaks have you had during your lessons?

What kinds of PA breaks would you like to have? Why?

How do PA breaks affect you?

How do PA breaks affect your studying and learning?
How did the PA break help you learn during this particular lesson? Describe closely what you mean.

Are breaks during lessons necessary in general upper secondary school? Motivate your answer.

In which way can PA breaks promote well-being among general upper secondary school students?

3) LEARNING ENVIRONMENT

Which lessons should include implemented PA breaks? Morning lessons? Afternoon lessons? Particular subjects?

How often should lessons include PA breaks?

At what point in the lesson should there be a PA break?

In which way should the sedentary time be cut, in your opinion?
